# Vaginally Assisted Natural Orifice Transluminal Endoscopic Surgery Hysterectomy for Giant Cervical Myoma: A Simple Procedure to Avoid Ureteral Injury

**DOI:** 10.1002/ccr3.71225

**Published:** 2025-10-12

**Authors:** Tomohiro Okuda, Arisa Egami, Wataru Suzuki, Yoko Uda, Shiho Sakai

**Affiliations:** ^1^ Department of Obstetrics and Gynecology Fukuchiyama City Hospital Fukuchiyama City Kyoto Japan

**Keywords:** hysterectomy, laparoscopy, myoma, natural orifice endoscopic surgery

## Abstract

Vaginally assisted natural orifice transluminal endoscopic surgery (vNOTES) hysterectomy has been compared to conventional laparoscopic total hysterectomy in previous reports. Incidentally, the risk of ureteral injury during total hysterectomy is lower with vaginal hysterectomy than with laparotomy or laparoscopy. Therefore, vNOTES hysterectomy, similar to vaginal hysterectomy, may be a safe option for total resection of cervical myomas.

**Summary**

- Total laparoscopic hysterectomy for giant cervical myoma growing within the retroperitoneal cavity may require the use of various techniques to avoid ureteral injury.
- Vaginally assisted natural orifice transluminal endoscopic surgery hysterectomy is a simple technique that involves elevating the uterus once the peritoneal vesico‐uterine fold and cul‐de‐sac are released.

## 1 Question

How are minimally invasive total hysterectomies for giant cervical myomas performed?

**FIGURE 1:**
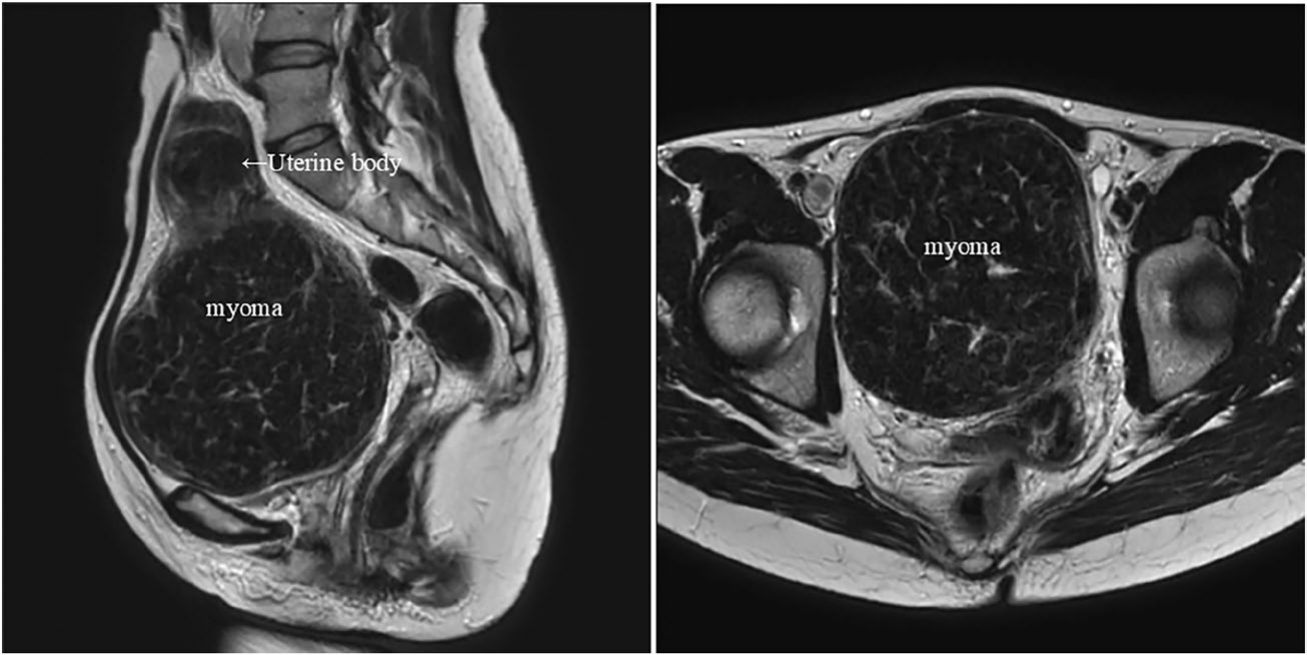
Magnetic resonance imaging scan of the 120‐mm cervical myoma and other myomas. Sagittal view of the uterine body (left) and axial view of the uterine myoma (right).

*Case*: A giant myoma (1110 g) was located in the right retroperitoneal cavity (Figure 1). MRI findings and laparoscopic manipulation are presented (Video 1). After anterior and posterior peritoneal release, only by pushing the cervix, the uterus was freed without complicated manipulations such as ureteric exposure or myomectomy [1]. The retractor (unlike power morcellators) facilitated vaginal dilation, allowing tissue extraction without spilling into the abdominal cavity [2].

**VIDEO 1:** First, the magnetic resonance imaging scan is presented. A giant cervical myoma was suspected to be growing in the right retroperitoneal space. Left‐sided and right‐sided procedures are presented. The left side of the giant uterus was protruding into the abdominal cavity. By simply pushing the cervix upwards, the left broad ligament could be easily dissected from the parametrial tissue to the ovarian ligament. The right side was embedded within the right retroperitoneal space. To perform the right‐sided procedure via conventional laparoscopic total hysterectomy, the course of the uterine artery and ureter must be identified, and this requires advanced technology. However, in vaginally assisted natural orifice transluminal endoscopic surgery hysterectomy since the strong uterine parametrial tissue was dissected, the embedded myoma could be easily detached by merely pushing the uterovaginal area upwards, and the right ovarian ligament was transected easily and quickly. Finally, the uterus retrieval process is presented. The vaginal canal was opened with a retractor, and the tissue was removed in one piece. Video content can be viewed at https://onlinelibrary.wiley.com/doi/10.1002/ccr3.71225.

## 2 Answer

Vaginally assisted natural orifice transluminal endoscopic surgery hysterectomy can be safely completed by moving the uterus intraperitoneally without exposing the ureter as the strong cervical ligaments are treated first.

## Author Contributions

**Tomohiro Okuda:** conceptualization, data curation, formal analysis, funding acquisition, funding acquisition, investigation, investigation, methodology, methodology, project administration, project administration, resources, resources, software, software, supervision, supervision, validation, validation, visualization, visualization, writing – original draft, writing – original draft, writing – review and editing, writing – review and editing. **Arisa Egami:** project administration, visualization, writing – review and editing. **Wataru Suzuki:** project administration, visualization, writing – review and editing. **Yoko Uda:** project administration, writing – review and editing. **Shiho Sakai:** project administration, writing – original draft, writing – review and editing.

## Ethics Statement

This report was approved by the Institutional Review Board at Fukuchiyama City Hospital (IRB No. 2024‐12‐45).

## Consent

Written informed consent was obtained from the patient.

## Conflicts of Interest

The authors declare no conflicts of interest.

## Data Availability

The data that support the findings of this study are available in the references listed at the bottom.
